# Evaluating the role of race and medication in protection of uterine fibroids by type 2 diabetes exposure

**DOI:** 10.1186/s12905-017-0386-y

**Published:** 2017-04-11

**Authors:** Digna R. Velez Edwards, Katherine E. Hartmann, Melissa Wellons, Anushi Shah, Hua Xu, Todd L. Edwards

**Affiliations:** 1 Vanderbilt Epidemiology Center, Vanderbilt University Medical Center, 2525 West End Ave., Suite 600 6th Floor, Nashville, TN 37203 USA; 2 Institute of Medicine and Public Health, Vanderbilt University Medical Center, Nashville, TN USA; 3 Vanderbilt Genetics Institute, Vanderbilt University Medical Center , Nashville, TN USA; 4 Department of Obstetrics and Gynecology, Vanderbilt University Medical Center, Nashville, TN USA; 5 Division of Diabetes, Endocrinology, and Metabolism, Department of Medicine, Vanderbilt University Medical Center, Nashville, TN USA; 6 Department of Biomedical Informatics, Vanderbilt University Medical Center, Nashville, TN USA; 7 The University of Texas School Health Science Center, School of Biomedical Informatics, Houston, TX USA; 8 Division of Epidemiology, Department of Medicine, Vanderbilt University Medical Center, Nashville, TN USA

**Keywords:** Uterine leiomyoma, Epidemiology, Electronic medical records, Gynecologic, Type 2 diabetes

## Abstract

**Background:**

Uterine fibroids (UF) affect 77% of women by menopause, and account for $9.4 billion in annual healthcare costs. Type-2-diabetes (T2D) has inconsistently associated with protection from UFs in prior studies. To further evaluate the relationship between T2D and UFs we tested for association between T2D and UF risk in a large clinical population as well as the potential differences due to T2D medications and interaction with race.

**Methods:**

This nested case–control study is derived from a clinical cohort. Our outcome was UF case-control status and our exposure was T2D. UF outcomes and T2D exposure were classified using validated electronic medical record (EMR) algorithms. Logistic regression, adjusted for covariates, was used to model the association between T2D diagnosis and UF risk. Secondary analyses were performed evaluating the interaction between T2D exposure and race and stratifying T2D exposed subjects by T2D medication being taken.

**Results:**

We identified 3,789 subjects with UF outcomes (608 UF cases and 3,181 controls), 714 were diabetic and 3,075 were non-diabetic. We observed a nominally significant interaction between T2D exposure and race in adjusted models (interaction *p* = 0.083). Race stratified analyses demonstrated more protection by T2D exposure on UF risk among European Americans (adjusted odds ratio [aOR] = 0.50, 95% CI 0.35 to 0.72) than African Americans (aOR = 0.76, 95% CI 0.50 to 1.17). We also observed a protective effect by T2D regardless of type of T2D medication being taken, with slightly more protection among subjects on insulin treatments (European Americans aOR = 0.42, 95% CI 0.26 to 0.68; African Americans aOR = 0.60, 95% CI 0.36 to 1.01).

**Conclusions:**

These data, conducted in a large population of UF cases and controls, support prior studies that have found a protective association between diabetes presence and UF risk and is further modified by race. Protection from UFs by T2D exposure was observed regardless of medication type with slightly more protection among insulin users. Further mechanistic research in larger cohorts is necessary to reconcile the potential role of T2D in UF risk.

**Electronic supplementary material:**

The online version of this article (doi:10.1186/s12905-017-0386-y) contains supplementary material, which is available to authorized users.

## Background

Uterine leiomyomata, or fibroids (UF), are the most common female pelvic tumor. Prevalence estimates for these benign growths range from 20 to 77%, increasing with age up to menopause [1–3]. The most well-established risk factors for UFs include African American race [1, 2, 4–7], high body mass index (BMI) [8, 9], and increasing age [4]. Suggestive protective associations have been observed in two studies, reducing relative risk of UFs by a third to a half [10, 11]. One of the studies evaluated the association among European Americans; however, they were underpowered, with only five T2D exposed UF cases and four controls. Neither study evaluated the potential interaction with race in the protective association between T2D and UFs. The protective association between T2D exposure and UFs was counterintuitive given the documented relationship between higher BMI and increased UF risk, but may be explained by a direct relationship between UFs and diabetes treatment.

A few studies support a potential relationship between UF risk, UF tumor growth, and diabetes treatment in modifying risk for UFs. The relationship with diabetes treatment was indirectly examined in one study among African Americans that found that the protection by diabetes was only among diabetics on medication (with medications incidence rate ratio [IRR] = 0.77, 95% confidence interval [CI] 0.60-0.98; without medications IRR = 0.91, 95% CI 0.64–1.28) [11]. Also supporting that the protection by diabetes derives from treatments are studies of tumorigenesis from in vivo and in vitro models of cancer reporting decreased risk of cancer among diabetics on medications, such as metformin, on a long term basis, usually greater than 5 years [12–15].

This study seeks to evaluate the relationship between type 2 diabetes exposure (T2D) and fibroid risk in large clinical cohort, including evaluating the role of diabetes treatments and race on risk for UFs. To obtain data for these analyses we used validated algorithms to classify cases and controls, as well as exposure to T2D from de-identified electronic medical records (EMR).

## Methods

### Study population

We utilized clinical data from the Synthetic Derivative (SD) database, located at Vanderbilt University, Nashville, TN [16]. The SD is a de-identified version of the EMR that consists of clinical data obtained from patients at all clinics in the Vanderbilt University Medical Center healthcare system. The SD is not a public database but is accessible with IRB approval to Vanderbilt University investigators. The SD contains several data types, including diagnostic and procedure codes, basic demographics, prescription medication information, imaging reports, discharge summaries, nursing notes, progress notes, health history, multi-disciplinary assessments, laboratory values, echocardiogram diagnoses, and electronically derived data. The Internal Review Board of Vanderbilt University, Nashville, TN approved this study and access to this SD database.

**Fig. 1:**
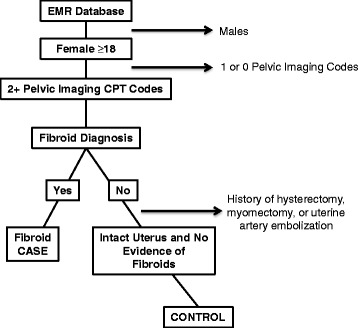
Fibroid case–control inclusion/exclusion criteria for nested case–control design. Provides a detailed description of the fibroid case–control inclusion/exclusion criteria

The outcome evaluated in these analyses was UF status after T2D evaluation. UF cases and controls (Fig. 1) were women at least 18 years old who had diagnostic imaging with ultrasound, magnetic resonance imaging (MRI), or computed tomography after evaluation for T2D. We excluded cases with an International Classification of Diseases 9^th^ edition [ICD 9] diagnostic code for UFs or current procedural terminology [CPT] code indicating UF removal prior to T2D evaluation. We included cases who had diagnostic imaging and either a diagnosis of a UF, as indicated by physician diagnosis, or a surgical procedure for UF removal after T2D evaluation. For controls, two or more instances of pelvic imaging on separate dates were required after T2D evaluation. Women with hysterectomy, myomectomy, or other procedures for UFs were excluded as controls. Our sampling algorithm to define UF cases and controls has been previously published [17] and is informed by a published UF algorithm by Hartmann and colleagues using EMRs [18]. This sampling algorithm results in 96% positive predictive value for cases, 98% negative predictive value for controls, 97% sensitivity, and 98% specificity. Information on covariate data was abstracted using natural language processing (NLP) algorithms of study participant EMRs, prescription medication information, as well as from ICD 9 diagnostic and CPT procedure codes. Prior validation studies of this phenotyping algorithm indicate that the majority of imaging information in both cases and controls comes from pregnancy ultrasounds [17].

T2D exposure was determined using a previously published and validated electronic algorithm that required diagnosis of T2D according to diagnostic codes, mentions of T2D medications in the medical record, and laboratory measures relevant to T2D diagnoses (glucose > 200 or HgbA1C > 6.5%) [19]. T2D controls were women who had at least 2 clinic encounters with one or more glucose or HgbA1C measures and who had no evidence of T2D diagnosis in their EMR such as text mentions or laboratory tests indicating a T2D diagnosis. This algorithm did not exclude based on Type I diabetes diagnostic codes due to those codes not being reliable in the EMR system. However, to confirm that individuals were not Type I diabetics we did a manual chart review of a subset of the records in our cohort that indicated a Type I diabetic code and in all cases confirmed T2D diagnosis. For a detailed description of the T2D algorithm refer to Ritchie et al. [19].

### Medication exposure assessment

T2D treatment information after diagnosis with T2D was extracted from both structured (e.g., computerized physician order entry) and unstructured (e.g., clinic visit notes) sources using MedEx, a high performance medication information extraction system [20, 21].

### Statistical methods

We performed logistic regression analyses to evaluate the association between T2D exposure and UF risk, unadjusted and adjusted for confounders. Potential confounders included in statistical models included age (years) at UF diagnosis for cases or last pelvic imaging for controls, body mass index (BMI) (kg/m^2^) at UF diagnosis or last pelvic imaging for controls, and race (European American [reference], African American, Hispanic, Asian, and Other). Secondary analyses were also performed evaluating effect modification by race, limiting to subjects who were African American or European American, using a likelihood ratio test. Finally, analyses were also performed limiting subjects to those on specific T2D medications including: metformin, thiazolidinedione, and insulin. We used a two-sided significance level of alpha = 0.05 for all tests of association. STATA statistical software version 11.0 (StataCorp LP, College Station, TX, USA) was used to perform tests and to prepare summaries of demographic characteristics. Effect sizes are presented as Odds Ratios (OR) with 95% confidence intervals (CIs).

## Results

There were 3,789 subjects included in analyses (Table 1). Mean age of UF diagnosis was 47 (standard deviation 13) and 44 (18) for controls. Women with UFs compared to those without UFs were more likely to be African American (35% T2D cases and 19% controls) and obese (BMI ≥ 30) (48% T2D cases and 31% T2D controls). There were slightly more women who were diagnosed with T2D among those without fibroids (UF cases with T2D 17% and controls with T2D 19%). HgbA1C levels were similar across UF cases and controls (data not shown). A detailed description of demographic characteristics by T2D exposure is provided in Additional file 1: Table S1.

**Table 1:** Study population characteristics and demographic variables by fibroid outcome

	n	Fibroid Cases (*N* = 608)	Fibroid Controls (*N* = 3,181)
		Mean(SD) or %	Mean(SD) or %
Age (years), mean (SD)	3,789	47(13)	44(18)
Race/ethnicity			
European American, non-Hispanic	2,502	56%	68%
African American, non-Hispanic	835	35%	19%
Hispanic ethnicity	73	2%	2%
Asian	45	1%	1%
Other	92	1%	3%
Missing	242	5%	7%
BMI (kg/m^2^), mean (SD)	2,496	34 (10)	32 (11)
Underweight (<20)	128	2%	4%
Normal weight (20–24)	468	11%	13%
Overweight (25–29)	610	21%	15%
Obese (≥30)	1,290	48%	31%
Missing	1,293	18%	37%
T2D diagnosis			
Yes (%)	714	17%	19%
No (%)	3,075	83%	81%
Diabetes treatments^a^			
Insulin	406	9%	12%
Metformin	360	11%	10%
Thiazolidinedione	133	5%	4%
Other medications	84	2%	3%

*BMI* body mass index, *SD* standard deviation

^a^Treatment percentages sum up to greater than 100% because women could have been on more than one treatment

Association analysis results between UF and T2D were modeled crude and adjusted for age, BMI, and race (Table 2). We tested for an interaction between race and T2D and observed a nominally significant (likelihood ratio test *p* = 0.083) interaction, and therefore also present results stratified by race. Since the interaction was only nominally significant we also report models adjusted for race. Race stratified analyses are limited to the largest racial groups, European American and African Americans, due to lower power for analyses among other racial groups. We observed an overall protective effect by T2D exposure on UF risk in both the overall adjusted model (adjusted OR [aOR] = 0.61, 95% CI 0.47 to 0.80) and race stratified models with a more protective effect observed among European Americans (aOR = 0.50, 95% CI 0.35 to 0.72) compared to African Americans (aOR = 0.76, 95% CI 0.50 to 1.17) (Table 2).

**Table 2:** Association of type II diabetes and fibroid risk

T2D Model	n	OR_T2D_	95% CI		P
			Lower	Upper	
**All Races**					
T2D	3,789	0.83	0.66	1.05	0.125
T2D, Age, BMI, Race	2,353	**0.61**	**0.47**	**0.80**	**2.73×10** ^**−4**^
**European American**					
T2D	2,502	**0.71**	**0.51**	**0.97**	**0.032**
T2D, Age, BMI	1,618	**0.50**	**0.35**	**0.72**	**2.07×10** ^**−4**^
**African American**					
T2D	835	0.94	0.65	1.37	0.749
T2D, Age, BMI	585	0.76	0.50	1.17	0.212

*T2D* type 2 diabetes, *BMI* body mass index, *OR* Odds ratio, *CI* 95% confidence interval; Bold indicates models with *p* < 0.05

Age and BMI are modeled as continuous measures

We also conducted secondary analyses stratifying diabetics by T2D medication and comparing them to controls in order to assess whether there were differences in UF based on the type of T2D medication (Table 3). These analyses were adjusted for age, BMI, and race, with secondary analyses stratifying by race. The effect size of T2D exposure on UFs was in the protective direction for all adjusted analyses evaluating the different types of medications, although not statistically significant in all analyses. However, the effect was most protective among those subjects who reported taking insulin for treatment of UFs in both the race adjusted (aOR = 0.50, 95% CI 0.35 to 0.70) and race stratified analyses, with a more protective effect among European Americans (aOR = 0.42, 95% CI 0.26 to 0.68) than African Americans (aOR = 0.60, 95% CI 0.36 to 1.01).

**Table 3:** Association analysis results for unadjusted and adjusted models of T2D exposure and UF risk limiting to subjects on specific treatments

	N	Crude				Adjusted			
		OR_T2D_	95% CI		P	OR_T2D_	95% CI		P
			Lower	Upper			Lower	Upper	
**All Races**									
T2D (diabetics on Metformin)	3,435	1.12	0.84	1.48	0.450	0.77	0.56	1.05	0.096
T2D (diabetics on Thiazolidinedione)	3,208	1.36	0.86	2.08	0.161	0.91	0.56	1.48	0.708
T2D (diabetics on insulin)	3,481	**0.71**	**0.52**	**0.97**	**0.032**	**0.50**	**0.35**	**0.70**	**5.10×10** ^**−5**^
**European American**									
T2D (diabetics on Metformin)	2,250	0.97	0.65	1.44	0.863	0.70	0.45	1.08	0.104
T2D (diabetics on Thiazolidinedione)	2,084	1.43	0.85	2.43	0.181	0.91	0.51	1.64	0.757
T2D (diabetics on insulin)	2,292	**0.60**	**0.38**	**0.95**	**0.020**	**0.42**	**0.26**	**0.68**	**3.54×10** ^**−4**^
**African American**									
T2D (diabetics on Metformin)	755	1.07	0.68	1.69	0.760	0.78	0.47	1.28	0.329
T2D (diabetics on Thiazolidinedione)	669	1.24	0.56	2.76	0.597	0.77	0.32	1.90	0.581
T2D (diabetics on insulin)	764	0.76	0.47	1.22	0.260	0.60	0.36	1.01	0.055

*T2D* type 2 diabetes, *BMI* body mass index, *OR* Odds ratio, *CI* 95% confidence interval; Bold indicates models with *p* < 0.05; Adjusted models use age (continuous), BMI (continuous); Bold indicates models with *p* < 0.05; N-samples size of total number of cases and controls within each drug category

To address the potential misclassification of T2D due to gestational diabetes due to the large number of the ultrasounds performed during pregnancies we conducted secondary analyses excluding subjects (*N* = 23) who were diagnosed with type 2 diabetes at the time of ultrasound or at the time (also diagnosis within 1 year before or after) a pregnancy diagnosis. This change had a very small effect on the effect sizes we estimated (data not shown), supporting that misclassification of chronic T2D status for transient gestational diabetes due to our study ascertainment criteria (requiring ultrasounds for inclusion as fibroid cases and controls) did not impact research findings significantly.

## Discussion

Consistent with prior studies, we observed protective associations between UFs and T2D in models adjusting for age, BMI, and race and in race stratified models. Race was observed to be an effect modifier for the association, although only nominally (*p* < 0.10), with slightly more protection by T2D exposure on UF risk among European Americans compared to African Americans. We evaluated models limited to women on specific T2D medications in secondary analyses. These models suggest that regardless of medication taken there was a protective effect of T2D exposure for developing UFs with slightly more protection among those taking insulin.

Diagnosis of diabetes has associated with protection from UFs in two studies, reducing relative risk of UFs by a third to a half [10, 11]. In our larger powered study we estimated effect sizes for analyses adjusting only for age and BMI (African American OR = 0.76, 95% CI 0.50 to 1.17; European American OR = 0.50, 95% CI 0.35 to 0.72) that were consistent with Baird and colleagues (African American OR = 0.67, 95% CI 0.35 to 1.29; European American OR = 0.96, 95% CI 0.24 to 3.76) and Wise and colleagues (African American diabetics on medication incidence rate ratio [IRR] = 0.77, 95% CI 0.60 to 0.98; diabetics not on mediations IRR = 0.97, 95% CI 0.64 to 1.28) who reported an inverse association between diabetes and UF. We note that Baird and colleagues was able to observe a suggestive association despite being underpowered (Baird and colleagues 35 diabetic cases and 19 diabetic controls). Additionally, all of the subjects we evaluated were being treated for diabetes; therefore, unlike Wise and colleagues, we were unable to evaluate the association using a diabetic population that was untreated.

Our findings suggested a protective effect by T2D exposure regardless of type of medication being taken, however, a slightly more protective effect was observed among those taking insulin. We were interested in the potential effect of T2D medications due to multiple studies showing that diabetic patients are at reduced risk of cancer if they are taking metformin and thiazolidinediones [12, 13, 15, 22–25]. The biological reasons why insulin may provide a slightly stronger protective effect is unclear, but may be due to the role of T2D severity in protection from fibroids, as women who are on insulin may have more severe forms of diabetes.

A single prior retrospective case–control study by Faerstein and colleagues evaluated the relationship between diabetic medications and UF risk and observed an increased risk for UFs among diabetics on medication [5]. This study also reported an effect size consistent with protection from UFs among diabetics when including all subjects in the cohort regardless of diabetes medication use (OR = 0.9, 95% CI 0.4 to 2.2). Cases were women with an ICD-9 218 code for UFs and confirmed by histologic findings or at least one ultrasound. Controls visited the same physician and had a prior pelvic examine without mention of findings consistent with having UFs. The study included 318 UFs cases and 394 controls, 4.7% of UF cases and 4.1% of controls were diabetic. Among diabetics only 2.2% of cases (*n* = 7) and 0.5% of controls (*n* = 2) reported being on a medication for diabetes. Although they were underpowered to detect an association, they observed a suggestive increased risk with OR = 2.1, 95% CI 0.4 to 12.6, adjusting for age, clinic, ethnicity, and BMI 5 years before interview. We note that differences in our findings and Faerstein and colleagues may be due to differences in power across these two studies as well as study design.

Our study has several strengths. We were able to leverage clinical EMR data to identify subjects diagnosed with UFs after T2D diagnosis. Furthermore, the utility of EMR combined with NLP approaches to abstract T2D medication use and detailed covariate data allowed us to comprehensively evaluate the relationship between UF and T2D medications. However, we acknowledge limitations to our study. In addition, although we evaluated T2D treatments that were initiated after diagnosis it is possible that some women were on these medications for other conditions that were not accounted for in these analyses. We also only evaluated whether or not a T2D treatment was initiated between T2D diagnosis and UF diagnosis but were unable to account for duration of treatment due to limitations of the study design. Finally, there is the possibility that another unaccounted for confounder like prior pregnancy history or hormonal treatments among these subjects may contribute to the observations observed.

## Conclusions

Our study supports prior studies that have observed a protection from UFs conferred by T2D diagnosis with somewhat more protection observed among European Americans than African Americans. Furthermore, our results suggest that the protective effect of diabetics occurs regardless of type of medication being taken, although subjects on insulin have slightly more protection from UFs. Furthermore, these data suggest that UFs have a complex etiology that involves interactions with multiple biological pathways. Further investigations are needed to determine what biologic mechanisms that may be involved in T2D pathways to protect from UFs.

## Additional file

Additional file 1: Table S1. Study population characteristics and demographic variables by T2D exposure. Provides a summary of study participant characteristics stratified by our primary exposure, type 2 diabetes. (DOCX 17 kb)

## Abbreviations

BMI: Body mass index
CI: Confidence interval
CPT: Current procedural terminology
EMR: Electronic medical records
ICD9: International Classification of Diseases 9^th^ edition diagnostic code
IRB: Institutional Review Board
IRR: Incidence rate ratio
MRI: Magnetic resonance imaging
NLP: Natural language processing
OR: Odds ratio
SD: Synthetic derivative
T2D: Type 2 diabetes
UF: Uterine fibroids
