# Supplementary material for: Evaluating the role of race and medication in protection of uterine fibroids by type 2 diabetes exposure
Source: BMC Womens Health. 2017 Apr 11;17:28. doi: 10.1186/s12905-017-0386-y (PMC5387248; doi:10.1186/s12905-017-0386-y)
Supplement: Additional file 1: Table S1. — Study population characteristics and demographic variables by T2D exposure. Provides a summary of study participant characteristics stratified by our primary exposure, type 2 diabetes. (DOCX 17 kb) [file 12905_2017_386_MOESM1_ESM.docx]

**Additional file 1: Table S1. Study population characteristics and demographic variables by T2D exposure**

|  | **n** | **T2D**  **(N = 714)** | **Non-Diabetics**  **(N= 3,075)** |
| --- | --- | --- | --- |
|  |  | **Mean(SD) or %** | **Mean(SD) or %** |
| Age (years), mean (SD) | 3,789 | 49 (17) | 44 (18) |
| Race/ethnicity |  |  |  |
| European American, non-Hispanic | 2,502 | 67% | 66% |
| African American, non-Hispanic | 835 | 26% | 21% |
| Hispanic ethnicity | 73 | 2% | 2% |
| Asian | 45 | 1% | 1% |
| Other | 92 | 2% | 2% |
| Missing | 242 | 2% | 8% |
| BMI (kg/m^2^), mean (SD) | 2,496 | 35(11) | 32(11) |
| Underweight (< 20) | 128 | 2% | 4% |
| Normal weight (20-24) | 468 | 12% | 12% |
| Overweight (25-29) | 610 | 15% | 16% |
| Obese (≥ 30) | 1,290 | 47% | 31% |
| Missing | 1,293 | 24% | 37% |
| Fibroid diagnosis |  |  |  |
| Yes | 608 | 14% | 16% |
| No | 3,181 | 86% | 84% |
| Diabetes treatments^1^ |  |  |  |
| Insulin | 406 | 57% | - |
| Metformin | 360 | 50% | - |
| Thiazolidinedione | 133 | 19% | - |
| Other medications | 84 | 12% | - |

BMI = body mass index; SD = standard deviation

^1^Treatment percentages sum up to greater than 100% because women could have been on more than one treatment.
